# Correction: Palladium nanoparticles immobilized on polyethylenimine-derivatized gold surfaces for catalysis of Suzuki reactions: development and application in a lab-on-a-chip context

**DOI:** 10.1039/d2ra90120j

**Published:** 2022-12-02

**Authors:** Prasad Anaspure, Subramanian Suriyanarayanan, Ian A. Nicholls

**Affiliations:** Linnaeus University Centre for Biomaterials Chemistry, Bioorganic and Biophysical Chemistry Laboratory, Department of Chemistry and Biomedical Sciences, Linnaeus University SE-39182 Kalmar Sweden subramanian.suriyanarayanan@lnu.se

## Abstract

Correction for ‘Palladium nanoparticles immobilized on polyethylenimine-derivatized gold surfaces for catalysis of Suzuki reactions: development and application in a lab-on-a-chip context’ by Prasad Anaspure *et al.*, *RSC Adv.*, 2021, **11**, 35161–35164. https://doi.org/10.1039/D1RA06851B.

The authors regret that the turnover numbers (TONs) were not correctly given in the original article.

In the abstract on page 35161, the corrected number should read 3.4 × 10^4^.

The corrected versions of Table 1 and 2 are shown below.

**Table 1:** Suzuki cross-coupling reactions of aryl halides with arylboronic acids using PEI/Pd as catalysts^a^

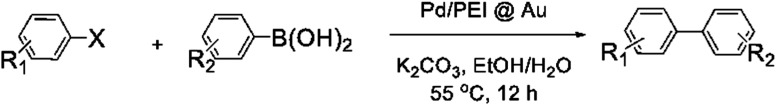						
Entry	R_1_	X	R_2_	Amount of Pd, μg	Yield	TON
1	H	I	H	3.2	93%	3.1 × 10^4^
2	H	Br	H	2.8	95%	3.4 × 10^4^
3	H	I	2-CH_3_	3.9	82%	2.2 × 10^4^
4	H	I	3-OCH_3_	4.0	57%	1.5 × 10^4^
5	H	I	4-OCH_3_	3.7	84%	2.4 × 10^4^
6	H	I	2-CN	3.99	15%	0.4 × 10^4^
7	H	I	4-CN	3.6	95%	2.8 × 10^4^
8	4-CH_3_	Br	H	6.2	88%	1.5 × 10^4^
9	4-OCH_3_	Br	H	8.4	95%	1.2 × 10^4^
10	H	I	H3–NH_2_	3.5	n. r.	—
11	H	Cl	H	1.0	94%	10.0 × 10^4^
12	4-OCH_3_	Cl	H	1.62	80%	5.3 × 10^4^
13	4-CoCH3	Cl	H	1.5	n. r.	—

a General procedure: 1.0 mmol of aryl halide, 1.2 mmol of arylboric acid, 2.0 mmol of k_2_CO_3_ in H_2_O/EtOH. Turnover number TON = mol product/mol Pd. n. r. = no reaction.

**Table 2:** Suzuki cross coupling reaction of iodobenzene and phenylboronic acid using PEI/Pd as catalyst^a^

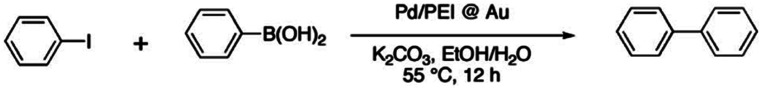				
Entry	Run	Conc. of Pd, αg	Yield	TON
1	1^st^	±2.3	93%	4.4 × 10^4^
2	2^nd^	±2.22	89%	4.3 × 10^4^
3	3^rd^	±2.22	85%	4.0 × 10^4^
4	4^th^	±2.15	80%	3.9 × 10^4^

a General procedure: 1.0 mmol of aryl halide, 1.2 mmol of arylboronic acid, 2.0 mmol of K_2_CO_3_ in H_2_O/EtOH. TON = mol product/mol.

Accordingly, Table 1-SI, Table 2-SI, Table 3-SI, and Table 4-SI in the original ESI have been revised; the ESI has been updated online.

An independent expert has viewed the corrected tables and has concluded that they are consistent with the discussions and conclusions presented.

The Royal Society of Chemistry apologises for these errors and any consequent inconvenience to authors and readers.

## Supplementary Material

